# Theoretical Modeling and Experimental Study on Low-Altitude Slow-Small Target (LSS) Detection Based on Broadband Spectral Modulation Imaging

**DOI:** 10.3390/s26030909

**Published:** 2026-01-30

**Authors:** Dongliang Li, Yangyang Hua, Siyuan Song, Jianguo Liu, Hongxing Cai

**Affiliations:** School of Physics, Changchun University of Science and Technology, Changchun 130022, China; 2022200010@mails.cust.edu.cn (D.L.); huajls@163.com (Y.H.); 2023100009@mails.cust.edu.cn (S.S.); liujianguo191019@126.com (J.L.)

**Keywords:** snapshot, spectral imaging, LSS target, wide-spectrum modulation

## Abstract

The detection of low-altitude slow-small (LSS) targets, such as drones, is challenged by their small radar cross-section (RCS) and low signal-to-clutter ratio (SCR), resulting in short effective range and susceptibility to background clutter in complex environments. To overcome the limitations of conventional radar and electro-optical methods, this paper proposes a novel detection theory based on broadband spectral modulation imaging (BSMI). We analyze the recognition accuracy for drone targets across different zenith angles and detection ranges through numerical simulations. A snapshot-based BSMI detection system was designed and implemented, with experiments conducted under consistent conditions for validation. Results demonstrate that the system achieves over 90% classification accuracy, confirming the theory’s effectiveness. This study significantly enhances detection probability and suppresses false alarms for low-altitude drones, providing a viable technical solution for monitoring unauthorized aerial activities.

## 1. Introduction

The detection of low-altitude, slow, and small (LSS) targets, including drones and other low-altitude aircraft, is of paramount importance for urban security, airspace management, and public safety [1]. With the rapid proliferation of drone technology for personal and commercial applications, there has been a significant increase in unauthorized flights, often referred to as “clandestine” or “unregulated” flights, particularly within restricted zones, posing serious challenges to urban governance [2]. Characterized by low observability, slow velocity, and low-altitude flight patterns, LSS targets substantially complicate detection efforts [3]. Consequently, the development of specialized detection systems capable of accurately identifying such targets is critically needed. These systems are essential for safeguarding citizen privacy, preventing illicit activities, and fostering the sustainable development of urban environments.

Current methodologies for detecting LSS targets primarily fall into three categories: acoustic, radar, and electro-optical (including visible and infrared) techniques. Acoustic detection identifies targets by analyzing their acoustic signatures, with studies by researchers such as Svanström, F and Zhang, T demonstrating effective drone recognition [4,5]. However, its utility is constrained to short ranges, typically under 200 m. Radar techniques, including Track-Before-Detect (TBD) and noise-whitening processes, leverage the Doppler effect to estimate kinematic and geometric target attributes [6,7]. Despite their robustness, radar systems are frequently susceptible to urban clutter and avian interference, resulting in elevated false alarm rates. In infrared detection, methods like two-dimensional least mean square (TDLMS) filtering and grayscale difference (GSD) algorithms have been employed to suppress background noise and enhance dim targets [8]. Nevertheless, the low thermal emissions characteristic of electric drones lead to poor thermal contrast with the environment, thereby reducing detection accuracy [9,10]. Visible-light detection outperforms infrared at close ranges and supports a spectrum of algorithms from traditional frame differencing to modern deep learning models [11,12,13,14]. Its performance, however, degrades over long distances due to atmospheric disturbances and occlusions. Collectively, each approach exhibits distinct limitations concerning operational range, false alarm control, or environmental adaptability, underscoring the pressing need for more robust LSS detection solutions.

Parallel to the evolution of conventional methods, spectral imaging technology has witnessed significant advancements in the realm of drone detection and tracking. Early research predominantly relied on traditional narrowband scanning or staring spectral imaging, which faced limitations in dynamic target recognition and long-range detection energy efficiency. Over the past five years, breakthroughs in snapshot spectral imaging and broadband modulation-demodulation technology have directed the field toward new developmental trajectories. In 2022, Wei Kai et al. utilized a liquid crystal tunable filter (LCTF) to analyze specific drone materials, achieving effective detection with dedicated algorithms [15]. In 2024, Sun et al. proposed a hyperspectral tracking algorithm for low-altitude drones that integrated deep learning with an improved kernel correlation filter, thereby enhancing both recognition accuracy and tracking efficiency [16]. Of particular note, broadband modulation-demodulation spectral imaging technology employs micro-nano structures such as metamaterials, quantum dots, or photonic crystals to achieve spectral encoding, endowing each pixel with unique broadband transmission characteristics and enabling highly efficient spectral information acquisition at the hardware level [17,18]. In 2025, Yu et al. demonstrated, for the first time, the identification of dynamic drone targets based on spectral features using snapshot spectral imaging, highlighting the application potential of this technology for low-altitude target detection [19]. These advancements signify a transition of spectral imaging from traditional fine spectral analysis towards novel detection systems that combine high energy efficiency, rapid response, and strong environmental adaptability, offering a new pathway for the robust detection of LSS targets against complex backgrounds.

The innovation of this work lies in the development of a snapshot spectral imaging system for drone target detection against complex backgrounds—such as clouds, forest canopies, and the sky—based on the rapidly advancing broadband modulation-demodulation spectral imaging technology. The primary contributions of this study are threefold:

First, systemic integration innovation. We have, for the first time, integrated a broadband modulation spectral chip [20] with a large field-of-view lens to construct a long-range LSS target detection system. This design overcomes the low energy efficiency of narrowband spectral detectors at long distances and mitigates the limitations of traditional scanning or staring spectral cameras in recognizing dynamic targets [10,21,22,23,24].

Second, enhanced detection mechanism and performance. By exploiting the distinct spectral differences among drones, interfering objects, and sky radiance [25,26,27,28], the proposed method effectively suppresses false alarms caused by cluttered backgrounds, enabling precise LSS target identification. Experimental results demonstrate that the system achieves both precision and recall rates exceeding 90% in real-world scenarios, representing a significant enhancement in overall detection performance.

Third, systematic evaluation of environmental adaptability. Through simulations and experimental tests, we have systematically assessed the impact of various zenith angles and atmospheric transmittance conditions on classification accuracy, providing crucial insights for the system’s deployment in diverse and variable practical environments.

This study not only validates the efficacy of broadband modulation-demodulation spectral imaging technology in addressing the challenges of long-range LSS target detection within complex backgrounds but also lays a key technological foundation for its engineering application and the advancement of intelligent perception capabilities in photoelectric systems.

## 2. Materials and Methods

### 2.1. Wide-Spectrum Modulation Spectral Imaging Detector: System Description

**Figure 1:**
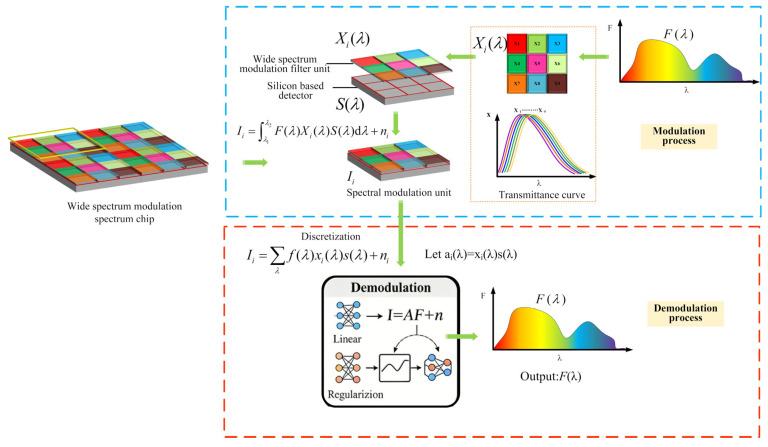
Schematic diagram of a wideband modulated imaging detector.

The wide-spectrum modulation spectral imaging detector, a key component in computational spectroscopy, is structured as shown in Figure 1. It integrates a wide-spectrum modulation filtering unit—composed of materials such as plasmonic filters [29,30,31], metasurfaces [32,33,34], or photonic crystal plates [35,36]—with a CMOS image sensor. Each filter unit modulates incident broadband light, overcoming the traditional compromise between energy throughput and spectral resolution in narrowband systems and thereby enhancing optical energy utilization. Assuming the incident spectrum is *F*(*λ*), and the transmission spectrum of each band-modulating filter unit is *X_i_*(*λ*), where *i* = 1, 2, 3, …, *N*, in Figure 1, *N* = 9, and *n_i_* represents the noise signal. The response curve of the CMOS image sensor is *S*(*λ*). The signal intensity value *I_i_* reaching each channel can be described as:

(1) $$I_{i}=\int_{\lambda_{1}}^{\lambda_{2}}F(\lambda )X_{i}(\lambda )S(\lambda )d\lambda +n_{i}$$

Applying discretization to the above equation yields its discrete form.

(2) $$I_{i}=\sum_{\lambda }f(\lambda )x_{i}(\lambda )s(\lambda )+n_{i}$$

Let *a_i_*(*λ*) = *x_i_*(*λ*)*s*(*λ*). The function *a_i_*(*λ*), which represents the combined system response, can be determined empirically by measuring its specific transmission spectrum. Consequently, the signal incident spectral irradiance *I* can be represented by a system of linear equations:

(3) I=AF(λ)+n

In this formulation, *I* ∈ R^*N*×1^, *A* ∈ R^*M*×*N*^, and *F*(*λ*) ∈ R^*M*×1^, with *N* being the number of wideband filter units and *M* the spectral sampling points. The case of *M* > *N* results in an overdetermined system that is often solvable, whsereas the underdetermined case (*M* < *N*) must be solved using regularization or data-driven methods like compressed sensing or convolutional neural networks.

In spatially modulated multispectral imaging systems, the inherently ill-posed nature of the system response matrix fundamentally limits spectral reconstruction quality. Directly applying traditional least squares methods yields solutions that are highly sensitive to noise, significantly compromising spectral accuracy. To mitigate this, we introduce a regularized least squares approach that incorporates appropriate constraints to embed prior knowledge into the inversion process. This strategy not only improves numerical stability and computational efficiency but also preserves spectral fidelity. By processing the spectral image across nine distinct bands, the proposed method achieves high-quality reconstruction of the target’s spectral information.

### 2.2. Calibration of the Wide-Spectrum Modulated Spectral Detector

The calibration of the wide-spectrum modulated spectral detector establishes the critical correspondence between incident radiation and sensor response, laying a theoretical foundation for accurate spectral reconstruction. The calibration system employs an optical configuration consisting of a bromine-tungsten lamp (400–2500 nm, Zolix, Beijing, China) coupled with a monochromator. Quasi-monochromatic light is generated across the detector’s operational band of 400–900 nm at 5 nm intervals. The light is homogenized by an integrating sphere before illuminating the detector surface, ensuring uniform field distribution. At each wavelength step, the digital responses of all modulation units are recorded simultaneously, thereby systematically acquiring the spectral transmission function of each filter element under controlled conditions.

Based on the complete dataset, the system response matrix **A** is constructed. Each column of the matrix corresponds to the normalized spectral response curve of a filter unit sampled at 5 nm resolution over the 400–900 nm range. This discrete representation quantitatively characterizes the modulation properties of the detector and forms the forward model for spectral encoding. Validation experiments using a standard reflectance white board were conducted, and the results were compared with measurements from a widely-used high-precision spectrometer (Ocean Optics QE65pro, Orlando, FL, USA). The Goodness of Fit Coefficient (GFC) exceeded 90%, demonstrating the system’s capability for high-accuracy spectral reconstruction across the 400–900 nm band. It should be noted that, for the purpose of LSS target detection in this study, spectral reconstruction does not aim for continuous high resolution over the full band. Instead, reconstruction is based on nine uniformly divided spectral segments—a level of accuracy that fully meets the requirements of the subsequent classification and identification tasks.

### 2.3. Theory of Small-Target Detection via Wide-Spectrum Modulation

The performance of long-range small target detection poses a critical dependence on irradiance conditions and atmospheric transmittance, which varies with season, latitude, and aerosol composition. The proposed theory utilizes the high optical throughput and spectral encoding capabilities of the wide-spectrum modulation detector to establish a spectral transfer equation. This equation models the target’s reflectance across the detector’s broadband channels, with its specific parameters defined below.

(4) $$\begin{array}{l} I_{1}(t,\lambda ,\theta )=\int_{\lambda_{1}}^{\lambda_{2}}sun(t,\lambda ,\theta )\cdot R(\lambda )\cdot AirT(t,\lambda ,\theta )\cdot LenT(\lambda )\cdot X_{1}(\lambda )\cdot S(\lambda )d\lambda +n_{1}\\ I_{2}(t,\lambda ,\theta )=\int_{\lambda_{1}}^{\lambda_{2}}sun(t,\lambda ,\theta )\cdot R(\lambda )\cdot AirT(t,\lambda ,\theta )\cdot LenT(\lambda )\cdot X_{2}(\lambda )\cdot S(\lambda )d\lambda +n_{2}\\ I_{3}(t,\lambda ,\theta )=\int_{\lambda_{1}}^{\lambda_{2}}sun(t,\lambda ,\theta )\cdot R(\lambda )\cdot AirT(t,\lambda ,\theta )\cdot LenT(\lambda )\cdot X_{3}(\lambda )\cdot S(\lambda )d\lambda +n_{3}\\ \vdots \\ I_{i}(t,\lambda ,\theta )=\int_{\lambda_{1}}^{\lambda_{2}}sun(t,\lambda ,\theta )\cdot R(\lambda )\cdot AirT(t,\lambda ,\theta )\cdot LenT(\lambda )\cdot X_{i}(\lambda )\cdot S(\lambda )d\lambda +n_{i} \end{array}$$

The key variable in the spectral transfer equation is *I_i_*(*t*,*λ*,*θ*), representing the signal intensity at the *I_i_*-th wideband modulation unit. It varies with time (expressed as seasonal/monthly parameter *t*), wavelength *λ*, and the solar zenith angle *θ*. The equation integrates several physical components: the illumination source *sun*(*t*,*λ*,*θ*), the target’s reflective property *R*(*λ*), the transmission losses through the atmosphere *AirT*(*t*,*λ*,*θ*) and the lens *LenT*(*t*,*λ*,*θ*), the spectral modulation *X_i_*(*λ*), the sensor response *S*(*λ*), and the noise *n_i_* per channel.

The target reflectance spectrum, when processed through the spectral transfer equation, produces a uniquely modulated signal across the nine broadband channels. This encoding strategy enhances the original spectral information and significantly increases detector sensitivity. Consequently, the system surpasses the detection range of traditional RGB and narrowband methods using the same image sensor. Thus, wideband modulation extends system range and improves recognition performance via optimized spectral processing.

**Figure 2:**
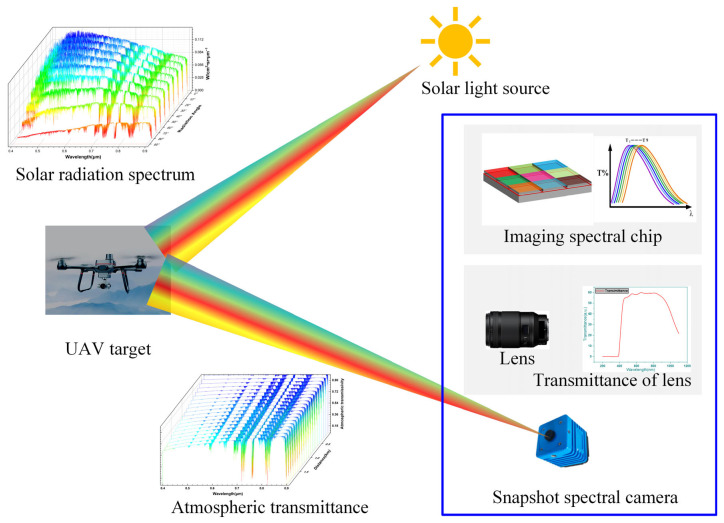
Theoretical flow of small target detection based on wideband modulation.

Figure 2 illustrates the complete imaging process of a UAV target captured by a snapshot spectral camera. Solar radiation incident upon the target generates a reflected spectral signal, which is then modulated by atmospheric transmittance before entering the camera system. The camera, comprising a lens assembly and a multispectral detector, further modifies this signal. As the light passes through the lens, its spectral profile is shaped by the lens’s optical transfer function and transmittance curve. This doubly modulated signal—by the atmosphere and the lens—is then projected onto the multispectral detector. There, it undergoes a third modulation by the spectral channel filter layers. Finally, photoelectric conversion at the silicon substrate yields the wide-spectrum modulated signal of the UAV target.

### 2.4. SVM-Based Classification and Evaluation Model for Small-Target Modulated Spectra

**Figure 3:**
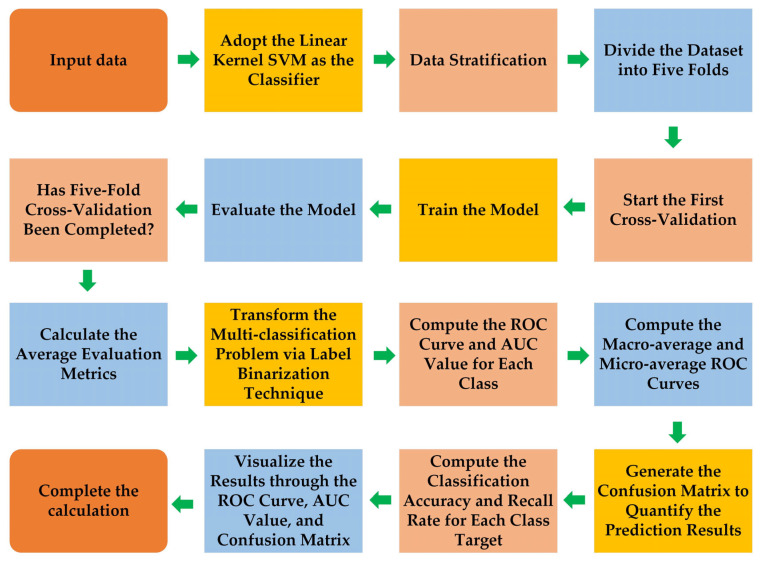
Process design of SVM classification and recognition algorithm.

Based on the previously established broadband modulation small target detection theory, this section employs Support Vector Machine (SVM) [31,37,38] to classify and identify UAV targets from background scenes (such as sky, clouds, birds, etc.). The input to the algorithm model is the nine-band spectral curve data of targets or backgrounds including UAVs, cloud layers, and birds. Firstly, spectral data of the targets and background are acquired within each modulation channel, followed by feature extraction from the spectral modulation signal curves per cycle. The extracted features are normalized using Z-score standardization to eliminate scale differences among feature dimensions, thereby enhancing data consistency and model robustness. The overall identification workflow based on the SVM algorithm is illustrated in Figure 3.

A linear kernel SVM was selected as the classifier for this spectral classification task, based on its computational efficiency and proven effectiveness with high-dimensional spectral features. To ensure robust performance evaluation, the dataset was partitioned using stratified k-fold cross-validation, which preserves the class distribution across all folds. Furthermore, the multi-class classification problem was decomposed into a set of binary tasks through a label binarization strategy, enabling the use of binary SVM classifiers for multi-class recognition.

To classify the preprocessed spectral features, a linear kernel SVM is employed. The algorithm aims to find an optimal decision hyperplane in the feature space that maximally separates the two classes by maximizing the classification margin—the minimum distance from any sample to the hyperplane. Under ideal conditions, SVM achieves perfect linear separation by minimizing the Euclidean norm of the weight vector, thereby enhancing generalization. In practical applications, however, a soft-margin strategy is adopted to handle non-separable cases and noise, introducing slack variables to allow some samples to violate margin constraints. This leads to an optimization problem that balances structural risk and empirical loss, regulated by a key parameter controlling the trade-off between model complexity and classification error. Algorithmically, SVM uses the hinge loss to linearly penalize misclassifications. Its convex optimization nature guarantees a global optimum, and the solution depends only on a subset of training samples called support vectors, providing desirable sparsity, computational efficiency, and strong generalization performance.

The classification performance is evaluated by calculating the ROC (Receiver Operating Characteristic) curves and AUC (Area Under the (ROC) Curve) values for each class, as well as computing the macro-average and micro-average ROC curves to comprehensively reflect the model’s overall performance. Additionally, to further analyze the model’s classification effectiveness, a confusion matrix is generated to quantify the prediction results, allowing for the calculation of classification accuracy and recall for each target class, thereby revealing the classifier’s recognition capability for different targets. The experimental results are visually presented through ROC curves, AUC values, and confusion matrices, providing an efficient analytical framework and performance evaluation basis for the spectral classification of small targets.

Using an SVM classifier to classify and recognize drone targets, sky, and white cloud modulated spectra, the model’s recognition accuracy is evaluated using metrics such as accuracy, recall, ROC curve, and confusion matrix.

Accuracy:

(5) $$\mathrm{Accuracy}=\frac{TP+TN}{TP+FP+FN+TN}$$

Recall:

(6) $$\mathrm{Recall}=\frac{TP}{TP+FN}$$

*TP* is a true positive example, *TN* is a true negative example, *FP* is a false positive example, and *FN* is a false negative example.

(7) $$\mathrm{TPR}=\frac{TP}{TP+FN}$$

(8) $$\mathrm{FPR}=\frac{FP}{TP+FN}$$

TPR is the true positive rate, and FPR is the false positive rate. The ROC curve shows the relationship between TPR and FPR at different thresholds, where the horizontal axis represents FPR and the vertical axis represents TPR, and the coordinate range is 0 to 1. The better the model performance, the closer its ROC curve is to the upper left corner and away from the diagonal line (the straight line from (0, 0) to (1, 1)). The AUC value represents the area under the ROC curve. The closer the value is to 1, the stronger the classification and recognition ability of the model.

## 3. Simulation: Setup, Results, and Analysis

### 3.1. Simulation Parameters and Setup

Based on the theoretical framework of wide-spectrum modulated small target recognition, a simulation scenario of a drone target against a sky background was constructed. The key simulation parameters comprised the reflectance spectrum of the drone material, the solar irradiance spectrum, atmospheric transmittance, and lens transmittance. A wide-spectrum modulation detector with nine distinct spectral channels was adopted for the detection process. The specific simulation parameters are summarized in the table below:

Based on the theoretical framework of wide-spectrum modulated small target recognition, a simulation scenario of a drone target against a sky background was constructed. The drone materials were selected from commonly used aerospace composites, including fiberglass, polypropylene, carbon fiber, and titanium alloy. The target scenario incorporated both sky and cloud backgrounds, while interference scenarios simulated typical forest occlusion conditions. For atmospheric transmission modeling, low-latitude summer aerosol parameters were adopted along with a rural visibility setting of 23 km. Solar radiation spectra were configured at zenith angles of 0° and 30°. Atmospheric transmittance curves and solar radiance spectra were simulated using MODTRAN, generating full-band spectral curves that account for atmospheric absorption and scattering effects. By integrating material properties, environmental parameters, and atmospheric data, physics-based simulations were performed using the Spectral Transfer Equation (Equation (1)) established in Section 2.2, ultimately producing modulated spectral datasets for both target and interference scenarios.

Noise is a key factor affecting the fidelity of simulations. To align with the detector used in experiments, a noise model based on actual hardware parameters was integrated into the simulation to improve reliability. The key detector parameters are as follows: a bit depth of 10 bits, a full-well capacity corresponding to a maximum digital number (DN) of 1023, and a measured dark noise standard deviation of approximately 64 DN. An additive Gaussian noise term was introduced to emulate practical signal degradation, with its amplitude varying dynamically—randomly fluctuating between 0% and 6% of the original signal value across channels—thereby more accurately representing the non-stationary noise characteristics under operational conditions.

### 3.2. Simulation Results and Discussion

Simulations were conducted under four representative scenarios, formed by combining solar zenith angles (0° and 30°) with detection distances (100 m and 200 m), using the parameters defined in Table 1. The target set included four common drone materials—fiberglass, polypropylene, carbon fiber, and titanium alloy—whose reflectance spectra were precisely measured with a standard spectrometer (Ocean QE65 Pro) to build a dedicated spectral library. To emulate realistic flight conditions, background and interference spectra were also incorporated, covering three typical categories: trees (terrestrial occlusion), blue sky (Rayleigh scattering), and white clouds (Mie scattering). Following the theory of wide-spectrum modulated point target detection, the spectral transfer equation was applied to simulate the system’s response, generating modulated channel values for each target, interference, and background spectrum. The resulting modulated spectral data for the four drone types were subsequently classified using a Support Vector Machine (SVM) algorithm. Final recognition performance was evaluated quantitatively through a confusion matrix and ROC curve.

**Figure 4:**
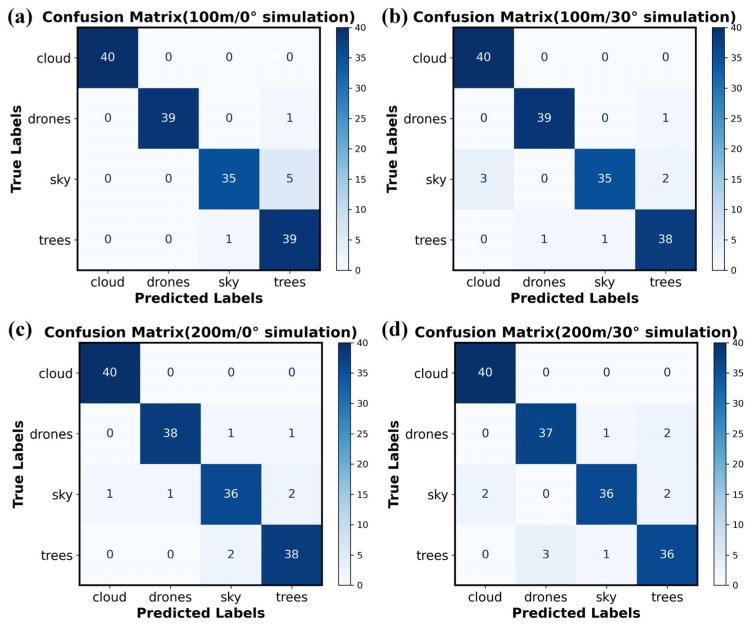
Confusion matrix of Simulation results for classifying and recognizing targets (e.g., drones, clouds, birds). (**a**) Simulation Results at 100 m Distance and 0° Zenith Angle. (**b**) Simulation Results at 100 m Distance and 30° Zenith Angle. (**c**) Simulation Results at 200 m Distance and 0° Zenith Angle. (**d**) Simulation Results at 200 m Distance and 30° Zenith Angle.

The classification performance of the SVM algorithm for the modulated spectra of drones, trees, clouds, and the sky was evaluated via confusion matrices. In each simulation scenario (defined by distance and zenith angle), a balanced dataset of 160 spectral samples (40 per target class) was used. Figure 4a presents the confusion matrix obtained under a solar zenith angle of 0° and a detection distance of 100 m, where rows denote true labels and columns represent predicted labels. Diagonal entries indicate correct classifications, with counts of 40 (clouds), 39 (drones), 35 (sky), and 39 (trees). Off-diagonal misclassifications include one drone identified as a tree, five sky samples as trees, and one tree sample as sky. Corresponding per-class accuracy values are 1.00, 0.975, 0.875, and 0.975, with recall rates of 1.00, 1.00, 0.972, and 0.86. These results confirm the effectiveness of the SVM-based classifier under the specified simulation conditions.

Further analysis of the confusion matrices under varying solar zenith angles and detection distances reveals distinct performance trends. As shown in Figure 4b, under a solar zenith angle of 30° and a detection distance of 100 m, the counts of correctly classified samples for clouds, drones, sky, and trees are 40, 39, 35, and 38, respectively. While all cloud samples are correctly identified, one drone is misclassified as a tree. For the sky category, three samples are mistaken for clouds and two for trees; in the tree category, one sample is misclassified as a drone and another as sky. The corresponding accuracy rates are 1.00, 0.975, 0.875, and 0.950, with recall rates of 0.93, 0.975, 0.972, and 0.926. These results indicate that as the solar zenith angle changes, the misclassification rate tends to increase, primarily due to altered solar spectral irradiance, which subsequently affects the modulation characteristics of the target reflection spectrum.

When the solar zenith angle is fixed at 0° and the detection distance extends to 200 m, the numbers of correctly classified samples for the four targets are 40, 38, 36, and 38, corresponding to accuracy rates of 1.00, 0.95, 0.90, and 0.95, and recall rates of 0.975, 0.974, 0.923, and 0.926, respectively. Compared with the 100-m case at the same zenith angle, classification accuracy declines for all targets except the sky category. This decrease is attributed to the increased influence of atmospheric transmittance on spectral band characteristics over longer distances. Nevertheless, as the detection range remains relatively short, overall classification performance remains satisfactory.

Under the most challenging simulated condition of 200 m detection distance and 30° solar zenith angle, the classification performance for all four target categories declined, with accuracy rates of 1.00, 0.925, 0.900, and 0.900, and recall rates of 0.952, 0.925, 0.947, and 0.900, respectively. All metrics stabilized around 0.9, demonstrating that the classification model maintains balanced and reliable accuracy even under degraded conditions. In summary, while variations in solar zenith angle and detection distance influence modulation spectrum classification, the model consistently sustains high accuracy within a reasonable operational range.

**Figure 5:**
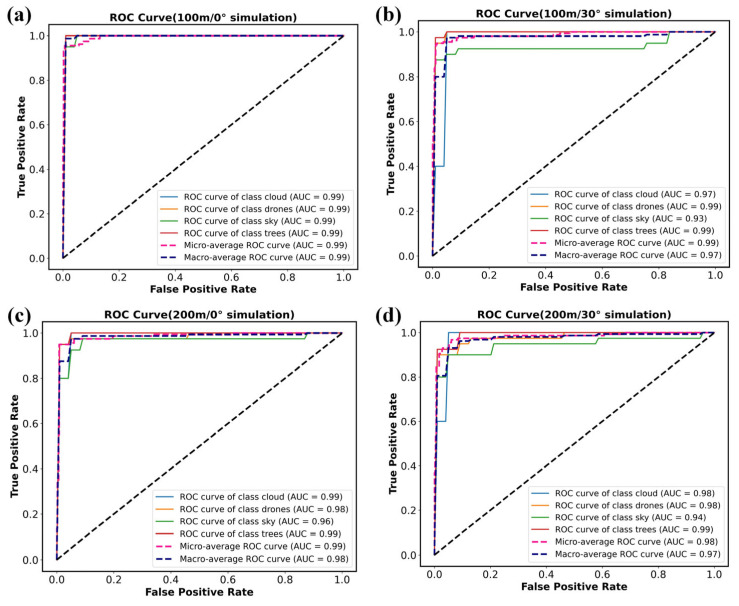
ROC curve of target classification and recognition simulation results at different zenith angles and detection distances. (**a**) ROC curve (100 m/0° Simulation). (**b**) ROC curve (100 m/30° Simulation). (**c**) ROC curve (200 m/0° Simulation). (**d**) ROC curve (200 m/30° Simulation).

Based on the classification results under different zenith angles and detection distances, the corresponding ROC curves were calculated, as shown in Figure 5a. At a solar zenith angle of 0° and a detection distance of 100 m, the AUC values for targets such as drones and trees reach 0.99. This indicates that the reflectance spectra of different targets exhibit distinct characteristics after modulation through the spectral transfer equation, and the SVM classifier effectively extracts these features, achieving excellent classification performance. When the distance increases to 200 m while the zenith angle remains 0°, the ROC curves shift slightly downward, yet the detection capability is not substantially degraded. Since detector noise is not included in the simulation, signal attenuation has limited impact on classification accuracy. When the distance is held constant and the zenith angle increases to 30°, the ROC curves for each target show modest fluctuations relative to the 0° case but remain close to the upper-left corner. Variations in solar illumination angle influence the modulation of target spectral features but do not cause a pronounced drop in classification accuracy. These results demonstrate that although the modulated spectral signal attenuates and fluctuates with increasing distance and zenith angle, the SVM model based on modulated spectral features maintains strong robustness and is well-suited for detecting LSS targets in complex environments.

## 4. Experimental Validation

### 4.1. Experimental System Setup

The developed snapshot-type LSS target detection system, based on wide-spectrum modulation, integrates three core components: a wide-spectrum modulation spectral chip, a wide-band large-field-of-view lens, and a 360° rotating mount. The spectral chip—a nine-channel imaging spectral detector fabricated by Changchun University of Science and Technology [10]—covers the 400–900 nm range with a pixel size of 1.8 μm × 1.8 μm and a spatial resolution of 1600 × 1200 pixels, supporting spectral image acquisition at 30 frames per second. The matched lens has a focal length of 2.48 mm, providing horizontal, vertical, and diagonal fields of view of 45.1°, 59.6°, and 76.5°, respectively. The entire optical assembly is mounted on a rotating bracket, enabling full-azimuth detection and identification of drone targets.

Upon completion of the system assembly, experimental validation was conducted under conditions consistent with the simulation. The tests were performed in a suburban environment with an atmospheric visibility of approximately 23 km. All experiments were carried out in June at a fixed detection distance of 100 m, with measurements taken at solar zenith angles of 0° (solar noon) and 30° (early afternoon). During testing, the system was securely positioned on the ground to capture drone targets against complex backgrounds including blue sky, white clouds, and woodland. To ensure adequate signal-to-noise ratio, imaging parameters were adjusted so that the average grayscale value of the spectral images remained between 180 and 220. Modulation spectrum data corresponding to the drone target, sky, and clouds were then extracted from the acquired images for subsequent analysis.

To ensure the precision consistency between experimental and simulation results, under stable experimental conditions, we conducted multiple tests on the scene data. Each test involved multiple captures of spectral images, which were then averaged to enhance the signal-to-noise ratio of the imagery. Furthermore, during the spectral reconstruction process, multiple samples were taken from different regions of the same target to ensure high accuracy in extracting the target spectrum.

**Figure 6:**
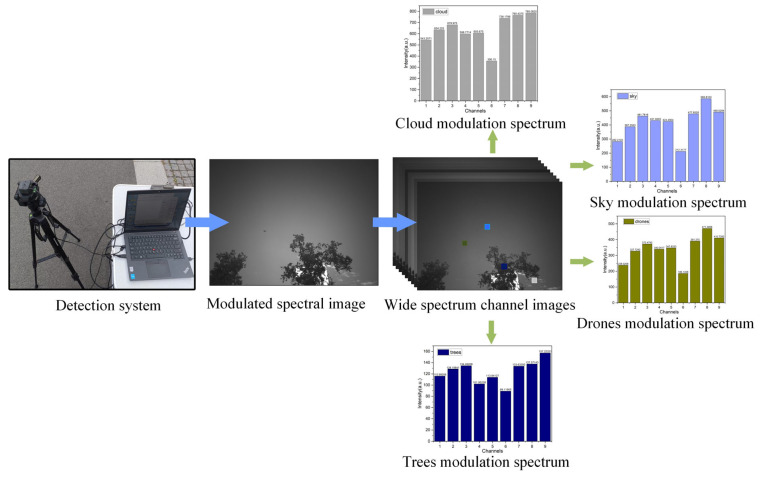
Schematic diagram of target modulation spectrum extraction.

Figure 6 details the process through which the detection system acquires modulated spectral information from aerial drone targets and background elements such as the sky, white clouds, and green vegetation. First, under set distance conditions, the system captures raw multispectral image data. This data reflects the response of the target within the periodic spectral modulation channels after being shaped by the system’s spectral transfer function. The acquired raw data is then processed to reconstruct spectral images for each modulation channel, producing multi-condition spectral representations of the target and its surrounding interference scenarios. Subsequently, gray values from selected regions of the modulated spectral data are extracted to derive their corresponding modulated spectral profiles. Experimental results reveal clear distinctions between the modulated spectral features of drone targets and background objects such as the sky, clouds, and vegetation. These differences provide a reliable data foundation and discriminative basis for subsequent target recognition and classification.

**Figure 7:**
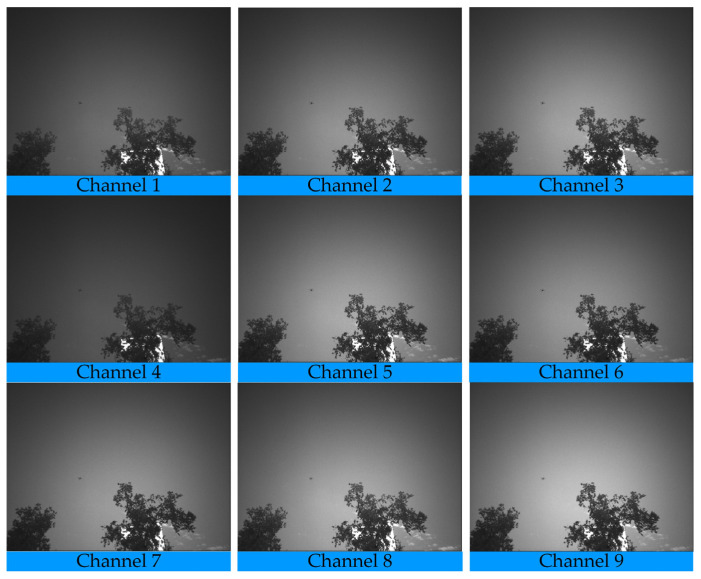
Modulated spectral images of different channels against the sky background.

Figure 7 presents the spectral images extracted from modulated data across different channels. Due to varying degrees of spectral modulation among channels, their grayscale values exhibit distinct profiles. Different elements within the scene—such as the drone target, sky background, white clouds, and forest—also show characteristic grayscale representations in each channel. Although the drone occupies only a small proportion of pixels in the image, it displays noticeable grayscale variation across multiple modulation channels, enabling it to be distinguished from background and other interfering objects. This result further confirms the feasibility and effectiveness of using target-modulated spectra for recognition and classification. On one hand, modulated spectra provide strong discriminability, effectively enhancing target-background contrast. On the other hand, the multispectral information from the nine channels offers richer spectral features, substantially improving the recognition capability and classification accuracy for typical targets such as drones. Consequently, modulated spectral-based multi-channel imaging demonstrates promising potential for detecting and identifying drone targets in complex backgrounds.

### 4.2. Experimental Results and Discussion

**Figure 8:**
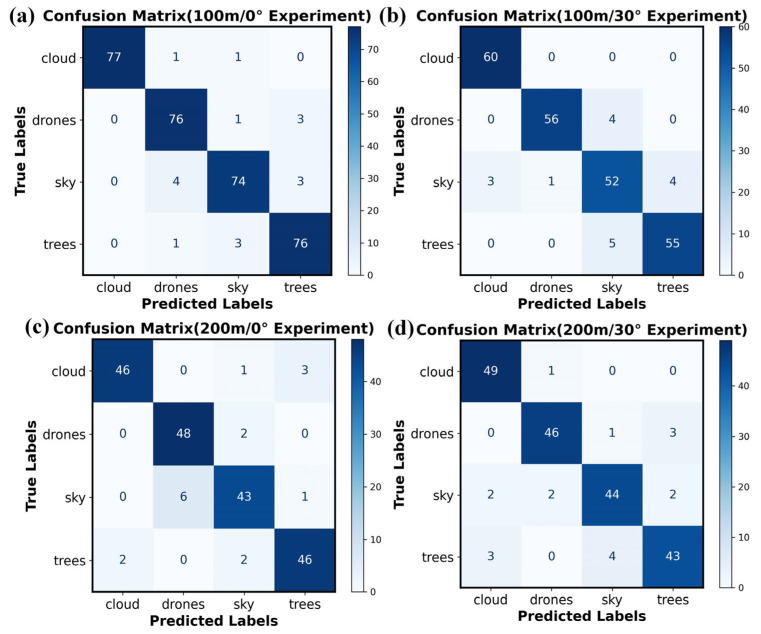
Confusion matrix of experimental results for classifying and recognizing targets (e.g., drones, clouds, birds). (**a**) Experimental Results at 100 m Distance and 0° Zenith Angle. (**b**) Experimental Results at 100 m Distance and 30° Zenith Angle. (**c**) Experimental Results at 200 m Distance and 0° Zenith Angle. (**d**) Experimental Results at 200 m Distance and 30° Zenith Angle.

Based on the foregoing simulation results, experimental validation was performed using the wide-spectrum modulation detector. In the experiment, the modulated spectral curves of targets were extracted from multi-scene spectral modulation images, and classification was conducted using an SVM algorithm with identical design parameters. The confusion matrix derived from the experimental results is shown in Figure 8. To ensure data randomness and experimental repeatability, datasets of different sizes were collected under varying environmental conditions, comprising 320, 240, 200, and 200 samples respectively. Each dataset contained an equal number of samples from the four target categories to maintain balance for the classification task.

Figure 8a presents the classification results for the four target categories: clouds, drones, sky, and trees. The numbers of correctly classified samples are 77, 76, 74, and 76, corresponding to accuracy values of 0.974, 0.95, 0.913, and 0.95, and recall rates of 1, 0.926, 0.936, and 0.926, respectively. For drone targets specifically, the accuracy and recall are 0.95 and 0.926. The primary factor affecting drone classification accuracy is the misclassification of sky samples as drones. This phenomenon is largely attributable to atmospheric transmittance and detector noise under long-distance testing conditions, which reduce the signal-to-noise ratio of drone targets in the modulated spectral images. In particular, at the edge regions of target pixels, the interference from sky background pixels diminishes the discriminability of the modulated spectral curve, leading to the misclassification of sky background as drone targets.

Further analysis was conducted with detection distance and solar zenith angle as key variables. When the detection distance remained fixed and the zenith angle increased to 30°, the overall classification accuracy for all targets decreased markedly, accompanied by a rise in misclassified samples. Notably, the recognition accuracy for drone targets declined further, with correct classifications being noticeably affected by interference from both the sky and trees. While in simulations solar radiation had limited influence on classification outcomes, in actual measurements the presence of detector noise altered the line shape of the target-modulated spectrum. This spectral distortion caused the modulated spectral signature of the drone to resemble those of the sky and trees more closely, thereby increasing classification errors.

When the solar zenith angle is 0° and the detection distance increases to 200 m, the classification accuracy for drones remains relatively stable, while the recall rate decreases to 0.88. This indicates an increase in false negatives, where samples of other categories are misclassified as drones. When both the detection distance and solar zenith angle change simultaneously, the overall classification accuracy for all targets declines to approximately 0.9, with drone classification accuracy reaching 0.92. These results demonstrate that under long-range detection conditions, the modulated spectral characteristics of targets such as drones are notably affected by atmospheric interference, leading to signal attenuation and reduced signal-to-noise ratio, which in turn significantly raises the misclassification and false alarm rates.

**Figure 9:**
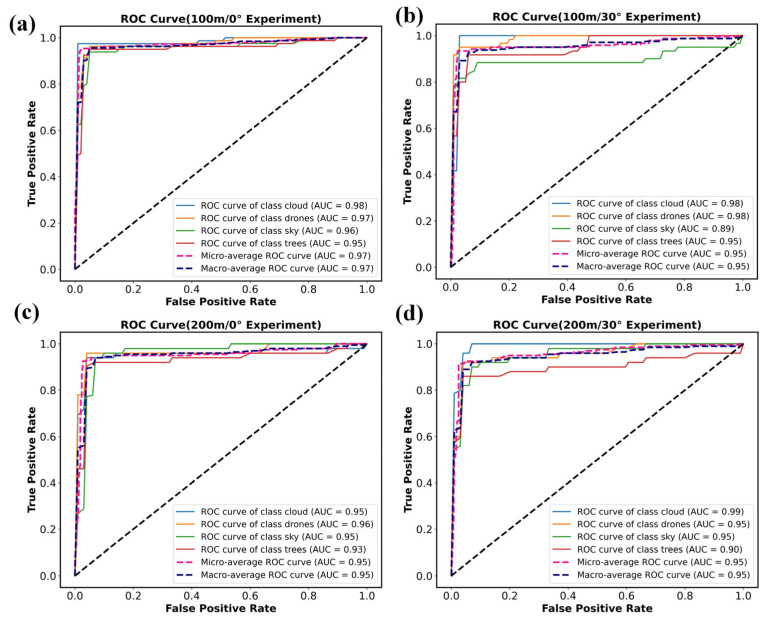
ROC curve of target classification and recognition simulation results at different zenith angles and detection distances. (**a**) ROC curve (100 m/0° Experiment). (**b**) ROC curve (100 m/30° Experiment). (**c**) ROC curve (200 m/0° Experiment). (**d**) ROC curve (200 m/30° Experiment).

As shown in Figure 9, the stability of the model was analyzed using ROC curves and AUC values, with classification performance evaluated across different detection distances, solar zenith angles, and target categories. The results indicate that the AUC values for drone targets remain consistently above 0.95, while the AUC values for the sky and trees exhibit greater variability, with minimum values of 0.89 and 0.90, respectively. Although the classification stability for the latter two categories shows some fluctuation, both the micro-average and macro-average AUC values across all categories exceed 0.95. This demonstrates that the model maintains robust overall classification performance and high recognition accuracy.

## 5. Discussion

### 5.1. Synthesis and Comparative Analysis of Simulation and Experimental Results

As shown in Table 2, the comparison between simulation and experimental results indicates that the simulated classification accuracy for targets such as drones exceeds the experimentally measured performance. Notably, the Root Mean Square Error (RMSE) between simulated and experimental outcomes for drones is the smallest among all targets, measuring only 0.026. The RMSE, defined as:

(9) $$\mathrm{RMSE}=\sqrt{\frac{1}{n}\sum_{i=1}^{n}{\left(Sim_{i}-Exp_{i}\right)}^{2}}$$

where *Sim_i_* and *Exp_i_* are the simulated and experimental values under the i-th condition, respectively, and *n* is the number of conditions, quantifies the average deviation between the two datasets. This low RMSE value reflects the closest agreement between simulation and experiment and confirms the higher recognition accuracy achieved by the model. This finding further substantiates the strong generalizability and practical validity of the wide-spectrum modulation theory for small target recognition, particularly in drone identification.

As shown in Table 3, the comparison of recall rates between simulation and experimental target recognition results indicates that for drone targets, the simulated recall ranges from 0.925 to 1, whereas the experimental recall ranges from 0.88 to 0.982. Although the experimental results are generally slightly lower than the simulation, they still maintain high accuracy. The RMSE between the two sets is 0.06, reflecting good agreement. However, relative to cloud and tree targets, the agreement between simulation and experiment is somewhat lower for drones. The primary reason is that extracting the modulated spectral curve from drone targets is more sensitive to image quality and the target’s pixel proportion in the scene. Especially under long-range detection, drone targets often occupy only tens or even fewer pixels, which increases the discrepancy between simulated and experimental outcomes.

### 5.2. Limitations of the Current Study and Future Perspectives

While this study has successfully established and validated a novel broadband modulation-demodulation framework for the detection of LSS targets, several limitations must be acknowledged, which also outline clear directions for future research. Firstly, the atmospheric model and noise injection methods employed in this work, although improved, remain relatively simplified. Future work will incorporate more complex, time-varying atmospheric profiles and a comprehensive noise model that includes fixed-pattern noise and nonlinear effects to enhance prediction fidelity. Additionally, while the current detection algorithm has proven effective, it has primarily been validated on a specific set of target types. Its generalization capability requires further evaluation across a broader spectrum of both LSS and non-LSS targets. Finally, systematic comparative analysis with other advanced spectral imaging techniques and deep learning-based classifiers is beyond the scope of this foundational study. Future research will focus on such benchmarking to quantitatively assess the advantages of the proposed technology. Addressing these limitations will advance the current proof-of-concept into a mature, field-deployable sensing solution.

Meanwhile, the current study primarily validates the detection performance under single or homogeneous backgrounds. Future work will focus on extending the research to various typical complex scenarios, such as urban and forest environments, while also considering the influence of different seasons, weather conditions, and lighting variations. This will allow for a systematic evaluation of the method’s generalization capability and environmental adaptability. Additionally, further efforts will be made to optimize the robustness of the algorithm in complex backgrounds, thereby advancing the technology toward practical engineering applications.

Finally, the current tests primarily focused on broadleaf trees as the vegetation target and cumulus clouds as the cloud target. The accuracy and generalizability of both simulation and experimental methods in more diverse scenarios require further improvement. Additionally, there are limitations in the current precision characterization of the data. In future work, we will continue to optimize the reflectance spectral database for different target categories and systematically enhance the generalization capability of the model in complex scenarios, thereby laying a foundation for the engineering application of this technology.

## 6. Conclusions

This study systematically presents a small-target detection method based on snapshot spectral imaging technology. Utilizing the operational principle of the wide-spectrum modulation spectral detector, the method comprehensively incorporates key factors in low-altitude slow-small (LSS) target detection, including material reflectance spectra, solar irradiance spectra, and atmospheric transmittance. Based on these factors, a theoretical framework for wide-spectrum modulated small-target recognition was established, followed by simulation analysis founded on the classification of small-target modulated spectra. Finally, a wide-spectrum modulation small-target detection system was constructed and tested on drone and woodland targets. Experimental results show that the recognition accuracy for drone targets lies between 0.92 and 0.95, with recall rates ranging from 0.88 to 0.982, aligning closely with simulation outcomes and confirming the effectiveness of this technical approach for LSS target detection. In practical detection, however, factors such as detector noise and limited dataset size can affect model performance, introducing fluctuations due to solar zenith angle and detection distance. Future work will focus on enhancing model stability and robustness for LSS target recognition by refining the noise term in the spectral transfer equation and expanding the training dataset.

## Figures and Tables

**Table 1:** Wide-spectrum modulation small target recognition simulation parameters.

Simulation Variables	Specific Parameters
drone material reflectivity spectrum	Glass fiber, polypropylene, carbon fiber, titanium alloy, etc.
Environment and interference target spectrum	Sky radiation spectrum, white cloud scattering spectrum, tree reflection spectrum
Atmospheric Transmittance Aerosol Selection	Rural-vis = 23 km
Irradiation type and zenith angle	Zenith angle 0°, 30°
Regional latitude	Low latitude
Seasons and dates	June (day180), Summer

**Table 2:** Comparative analysis of the accuracy of simulation and experimental target classification and recognition results.

	Sim (Drones)	Exp (Drones)	Sim (Cloud)	Exp (Cloud)	Sim (Sky)	Sim (Sky)	Sim (Trees)	Exp (Trees)
(0°, 100 m)	0.975	0.95	1	0.974	0.875	0.913	0.975	0.95
(0°, 200 m)	0.95	0.96	1	0.92	0.9	0.86	0.95	0.92
(30°, 100 m)	0.975	0.93	1	1	0.875	0.88	0.95	0.916
(30°, 200 m)	0.925	0.92	1	0.98	0.9	0.878	0.9	0.86
RMSE	0.026		0.043		0.03		0.052	

**Table 3:** Comparative analysis of recall rates of simulation and experimental target classification and recognition results.

	Sim (Drones)	Exp (Drones)	Sim (Cloud)	Exp (Cloud)	Sim (Sky)	Sim (Sky)	Sim (Trees)	Exp (Trees)
(0°, 100 m)	1	0.926	1	1	0.972	0.936	0.866	0.926
(0°, 200 m)	0.974	0.88	0.975	0.95	0.923	0.89	0.926	0.92
(30°, 100 m)	0.975	0.982	0.93	0.952	0.972	0.852	0.926	0.932
(30°, 200 m)	0.925	0.938	0.952	0.907	0.947	0.897	0.9	0.895
RMSE	0.06		0.028		0.069		0.03	

## Data Availability

The original contributions presented in this study are included in the article. Further inquiries can be directed to the corresponding author.
